# Prevalence of multidrug-resistant and extended-spectrum beta-lactamase producing Gram-negative isolates from clinical samples in a tertiary care hospital of Nepal

**DOI:** 10.1186/s41182-021-00313-3

**Published:** 2021-03-11

**Authors:** Aryatara Shilpakar, Mehraj Ansari, Kul Raj Rai, Ganesh Rai, Shiba Kumar Rai

**Affiliations:** 1 Shi-Gan International College of Science and Technology (SICOST), Tribhuvan University, Kathmandu, Nepal; 2 Institute of Microbiology, University of Chinese Academy of Sciences, Beijing, China; 3 Nepal Medical College, Kathmandu, Nepal

**Keywords:** MDR, ESBL, Gram-negative isolates, Carbapenems, Polymyxin B, Nepal

## Abstract

**Background:**

The existence of multidrug-resistant organisms, including extended-spectrum beta-lactamases (ESBLs), is on rise across the globe and is becoming a severe problem. Knowledge of the prevalence and antibiogram profile of such isolates is essential to develop an appropriate treatment methodology. This study aimed to study the prevalence of Gram-negative isolates exhibiting ESBL at a tertiary care hospital and study their antibiogram profile.

**Methods:**

A cross-sectional study was conducted at Shahid Gangalal National Heart Centre, Kathmandu, Nepal, from June 2018 to November 2018. A total of 770 clinical samples were collected and identified using the conventional biochemical tests following the Clinical and Laboratory Standard Institute (CLSI) guidelines. Antimicrobial susceptibility testing (AST) was performed using the standardized Kirby-Bauer disk diffusion method. The screening test for ESBL producers was performed as recommended by the CLSI and the confirmatory test was performed phenotypically using the E-test.

**Results:**

Out of the 92 isolates, 84 (91.3%) were multidrug-resistant, and 47 (51.1%) were found to be potential ESBL producers. Of these, 16 isolates were confirmed ESBL producers by the E-test. *Escherichia coli* and *Klebsiella pneumoniae* were the predominant isolates and were also the major ESBL producers. Besides polymyxin B (100% sensitive), meropenem and imipenem showed high efficacy against the ESBL producers.

**Conclusion:**

Multidrug resistance was very high; however, ESBL production was low. Polymyxin B and carbapenems are the choice of drugs against ESBL producers but should be used only as the last line drugs.

## Background

Although advancements in medical technology continue to grow, the combat against drug-resistant microbes is always challenging and drug resistance is widely considered to be the next global pandemic [1]. Bacteria have been ever-evolving and conferring resistance to advanced and more powerful antibacterial drugs. For instance, extended-spectrum beta-lactamase (ESBL) producers can confer resistance to beta-lactam antibiotics such as penicillins, cephalosporins, and aztreonam [2]. Moreover, ESBL producers often carry genes responsible for resistance to antimicrobial even other than beta-lactamases [3]. Therefore, ESBLs producers exhibit a broad antibiotic resistance which is a great problem in clinical therapeutics [3–5] and leads to higher morbidity and mortality owing to improper or delayed antibiotic treatment [6]. ESBLs are becoming more common in Gram-negative isolates [7, 8], and the number of ESBL producers is growing exponentially [9]. Many new classes of β-lactam antibiotics have been developed over the years, but the overuse of such antibiotics has resulted in the emergence of new variants of β-lactamases [10]. Hence, ESBL producers have become a major multidrug-resistant pathogen, and several significant changes in ESBL producing isolates have been witnessed worldwide in the last two decades [11].

Every year, an estimated 23,000 deaths in USA and 25,000 deaths in the EU are caused by multidrug-resistant (MDR) bacterial infection [12]. There is limited information regarding mortality attributed by MDR bacterial infection in developing most countries like Nepal. Prevalence of MDR and ESBL producing isolates has been reported to be on rise worldwide, and most of the studies showed increasing trend of prevalence in recent times [13, 14]. South Asia has been reported as a key spot for the incidence of antimicrobial-resistant pathogens, with a high prevalence of MDR and ESBL [15–18]. The problem is most noticeable in developing countries like Nepal [1, 12], and the prevalence of MDR and ESBL producing pathogen is as higher as 90% in Nepal, as evidenced from the recent past studies [19–22]. Widespread irrational and injudicious use of high doses of antibiotics, easy availability of antibiotics, and the practice of self-medication without prescription, prescription even before the AST results are some of the forms of antibiotic misuse in Nepal and responsible for high antibiotic resistance in Nepal [23, 24]. Clinicians frequently face difficulty selecting suitable empirical therapy for infections caused by MDR and ESBL producing isolates [25]. Clinicians need to be familiar with the clinical significance of ESBLs and potential strategies for dealing with them [26]. The knowledge of local epidemiology and few other factors helps in choosing the best antibiotic therapy [25]. Regular surveillance of MDR and ESBL production is needed to guide appropriate antimicrobial therapy [27] and minimize the risk of developing resistance with certain drugs soon [28]. Thus, we conducted a study to determine the prevalence and antimicrobial susceptibility pattern of ESBL producing Gram-negative bacteria isolated from different clinical samples at tertiary care hospitals.

## Methods

### Study design

A hospital-based cross-sectional study was conducted for 6 months (June to November 2018) at Shahid Gangalal National Heart Centre, Kathmandu, Nepal. Different clinical samples taken from patients admitted in the hospital and from visiting outpatient departments of the hospital were included. In the case of urine and sputum, proper information about sample collection was given. Improperly collected samples or those lacking proper labeling were excluded from the study.

### Isolation and identification of the isolates

A total of 770 samples (urine, 250; sputum, 185; pus, 115; blood, 166; wound swab, 49; and tissue, 5) were processed (cultured) following the Clinical and Laboratory Standard Institute (CLSI) guidelines. The specimens were cultured on nutrient agar, brain heart infusion (BHI) broth (only for blood samples), MacConkey agar, and blood agar. The isolates were identified based on colony morphology, Gram’s stain result, and conventional biochemical methods [29].

### Antimicrobial susceptibility testing

Antimicrobial susceptibility testing (AST) was done by the Kirby-Bauer disk diffusion technique using Muller Hinton agar (MHA) [30, 31]. The antibiotics used were amikacin (30 μg), amoxyclav (30 μg), amoxicillin (10 μg), gentamicin (10 μg), ceftriaxone (30 μg), norfloxacin (10 μg), cotrimoxazole (25 μg), ciprofloxacin (5 μg), cefixime (30 μg), gentamicin (10 μg), nitrofurantoin (30 μg), nalidixic acid (30 μg), ofloxacin (5 μg), meropenem (10 μg), piperacillin/tazobactam (100/10), polymyxin B (100 IU), imipenem (10 μg), and cefepime (30 μg). The bacterial isolates showing resistance towards three or more different antibiotics classes were considered multidrug-resistant (MDR) bacteria.

### Screening and confirmation of ESBL producers

Ceftazidime, cefpodoxime, ceftriaxone, and cefotaxime were included in the primary panel for screening potential ESBL producers. Isolates showing resistance to any of these antibiotics were suspected as potential ESBL producers and were confirmed by E-test (Ezy MIC^TM^). For this, a lawn culture of the test organism was done on MHA which the Ezy MIC^TM^ strip was applied using the applicator, and reading was taken after incubation. Minimum inhibitory concentration (MIC) value (where the edge of the inhibition ellipse intersects the strip’s side) was noted. ESBL production was confirmed as positive when the ratio of the MIC value obtained for ceftazidime or ceftriaxone combined with the clavulanic acid (CAZ+ or CTR+) was more than 8 or when no zone obtained for CTR or CAZ and zone obtained in CTR+ or CAZ+ [32].

### Quality control

All batches of the culture media and chemical reagents were processed with aseptic techniques following CLSI guidelines applying a standard aseptic procedure. In AST, quality control was maintained by using the control strains of *E*. *coli* ATCC 25922. Quality control of Ezy MIC™ strip was carried out by testing the strips with standard ATCC strains (*E*. *coli* ATCC 25922 and *Klebsiella pneumoniae* ATCC 700603).

### Data analysis

SPSS v16.0 was used for statistical analysis. Chi-square test was applied at 95% CI among demographic variables.

## Results

A total of 92 Gram-negative bacteria were isolated from various clinical specimens. *K*. *pneumoniae* (*n* = 35) and *E*. *coli* (*n* = 29) were the predominant isolates. Highest number of the organism was isolated from urine (*n* = 52) followed by sputum (*n* = 18) (Table 1). A total of 39 and 50 organisms were isolated from the inpatient department and outpatient department, respectively. The highest number of organisms was isolated from the age-group above 60 and the least from the age-group 11–20 (Table 3). There was no significant difference in growth positivity between males and females (*p* = 0.134); however, growth positivity in urine was significantly higher in females (77.3%) than in males (37.5%) (*p* = 0.012).

**Table 1:** Sample-wise distribution of isolates

Microorganisms	Specimens						Total
	Urine	Sputum	Pus	Blood	Wound	Tissue	
*E*. *coli*	28	0	0	1	0	0	29
*K*. *pneumoniae*	15	11	3	2	2	2	35
*P*. *aeruginosa*	1	3	0	2	0	0	6
*Acinetobacter* spp.	5	4	1	3	1	0	14
*Enterobacter* spp.	1	0	0	0	1	0	2
*P*. *mirabilis*	2	0	0	0	0	0	2
*S*. *marcescens*	0	0	2	0	1	1	4
**Total**	**52**	**18**	**6**	**8**	**5**	**2**	**92**

### Antibiotic susceptibility pattern of Gram-negative isolates

Of the 20 different antibiotics used, polymyxin B was the most effective drug (100% sensitivity), whereas amoxicillin (3.3% sensitivity) was the least effective (Table 2). In AST, 47 (51.1%) isolates showed resistance to one or more cephalosporin used and were suspected to be ESBL producers.

**Table 2:** Organism-wise antibiotic resistance pattern

Antibiotics	Isolates						
	***E***. ***coli*** (***n*** = 29)	***K***. ***pneumoniae*** (***n*** = 35)	***P***. ***aeruginosa*** (***n*** = 6)	***Acinetobacter*** (***n*** = 14)	***Enterobacter*** (***n*** = 2)	***P***. ***mirabilis*** (***n*** = 2)	***S***. ***marcecens*** (***n*** = 4)
**Amoxycillin**	100.0%	94.3%	100.0%	100.0%	100.0%	100.0%	75.0%
**Amikacin**	34.5%	77.1%	50.0%	78.6%	100.0%	0.0%	50.0%
**Cipofloxacin**	96.6%	88.6%	66.7%	100.0%	0.0%	100.0%	50.0%
**Cotrimoxazole**	65.5%	60.0%	100.0%	100.0%	100.0%	50.0%	50.0%
**Gentamicin**	27.6%	80.0%	83.3%	78.6%	0.0%	50.0%	0.0%
**Malidixic acid**	100.0%	97.1%	100.0%	92.9%	100.0%	100.0%	25.0%
**Polymyxin B**	0.0%	0.0%	0.0%	0.0%	0.0%	0.0%	0.0%
**Ofloxacin**	96.6%	88.6%	66.7%	100.0%	0.0%	100.0%	50.0%
**Amoxyclav**	79.3%	85.7%	100.0%	92.9%	100.0%	100.0%	0.0%
**Ceftriaxone**	86.2%	91.4%	83.3%	92.9%	0.0%	0.0%	50.0%
**Cefixime**	100.0%	91.4%	100.0%	100.0%	0.0%	0.0%	25.0%
**Ceftazidime**	89.7%	91.4%	83.3%	100.0%	100.0%	0.0%	50.0%
**Piperacillin tazobactam**	20.7%	77.1%	50.0%	78.6%	0.0%	50.0%	0.0%
**Tobramycin**	44.4%	80.0%	40.0%	78.6%	-	-	-
**Cefepime**	93.1%	91.4%	50.0%	85.7%	100.0%	50.0%	25.0%
**Meropenem**	24.1%	60.0%	50.0%	64.3%	0.0%	0.0%	0.0%
**Imipenem**	20.7%	57.1%	50.0%	78.6%	0.0%	0.0%	25.0%
**Nitrofurantoin**	24.1%	74.3%	-	85.7%	100.0%	100.0%	50.0%
**Norfloxacin**	96.6%	88.6%	66.7%	100.0%	0.0%	100.0%	50.0%
**Cephodoxime**	93.1%	100.0%	83.3%	78.6%	100.0%	100.0%	50.0%

### Antibiotic susceptibility pattern of ESBL producers

**Fig. 1:**
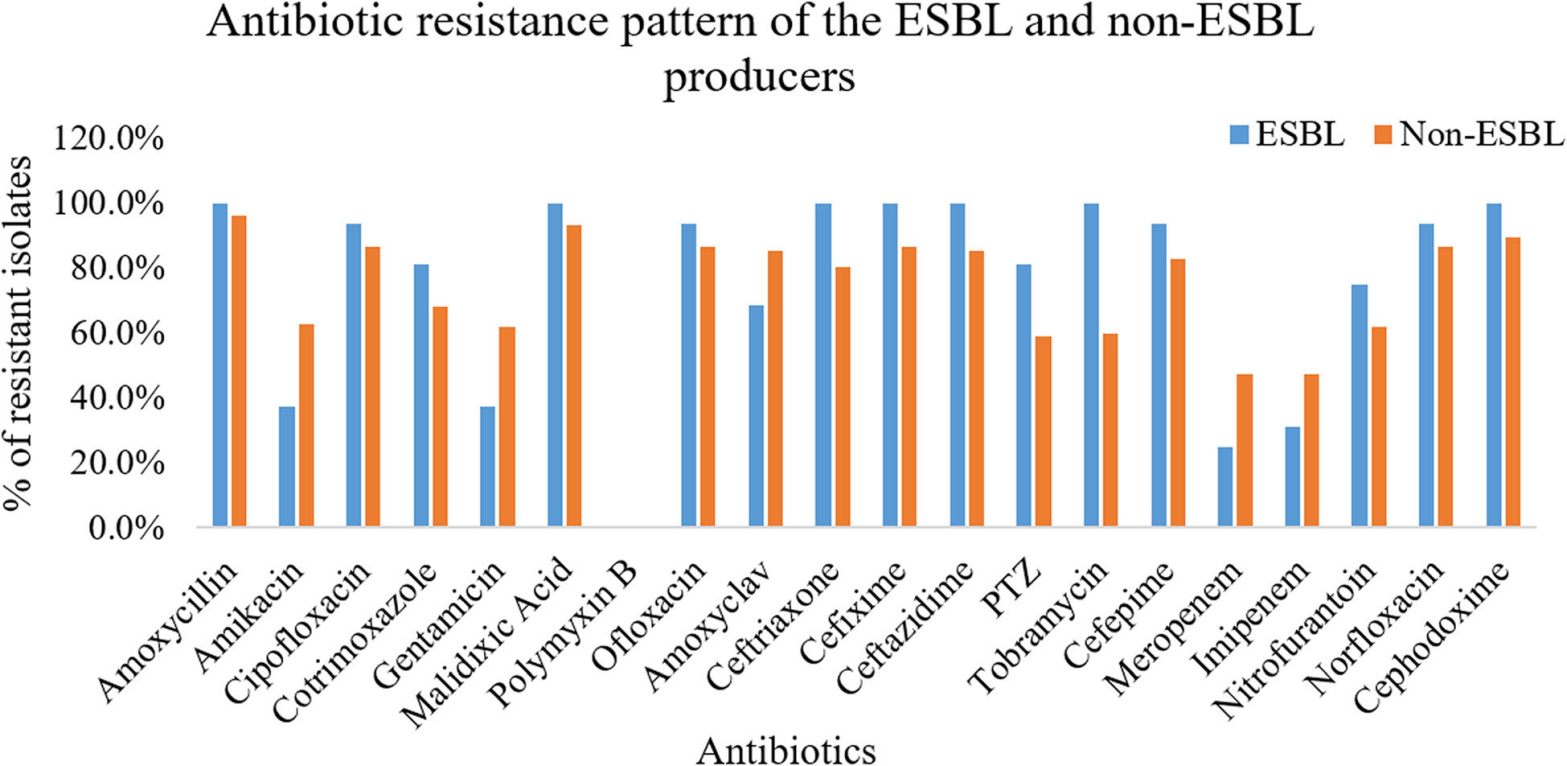
Antibiotic resistance pattern of the ESBL and non-ESBL producers

Of the 20 different antibiotics used against Gram-negative bacteria, polymyxin B showed 100% effectiveness to ESBL producers. Meropenem and imipenem were sensitive to around three fourths of the ESBL producers. Amoxycillin and gentamicin were also effective against more than three fifths of the ESBL producers. All other antibiotics used showed reduced sensitivity to the ESBL producers as compared to the non-ESBL producing isolates (Fig. 1).

### Distribution of ESBL producers in Gram-negative isolates

**Fig. 2:**
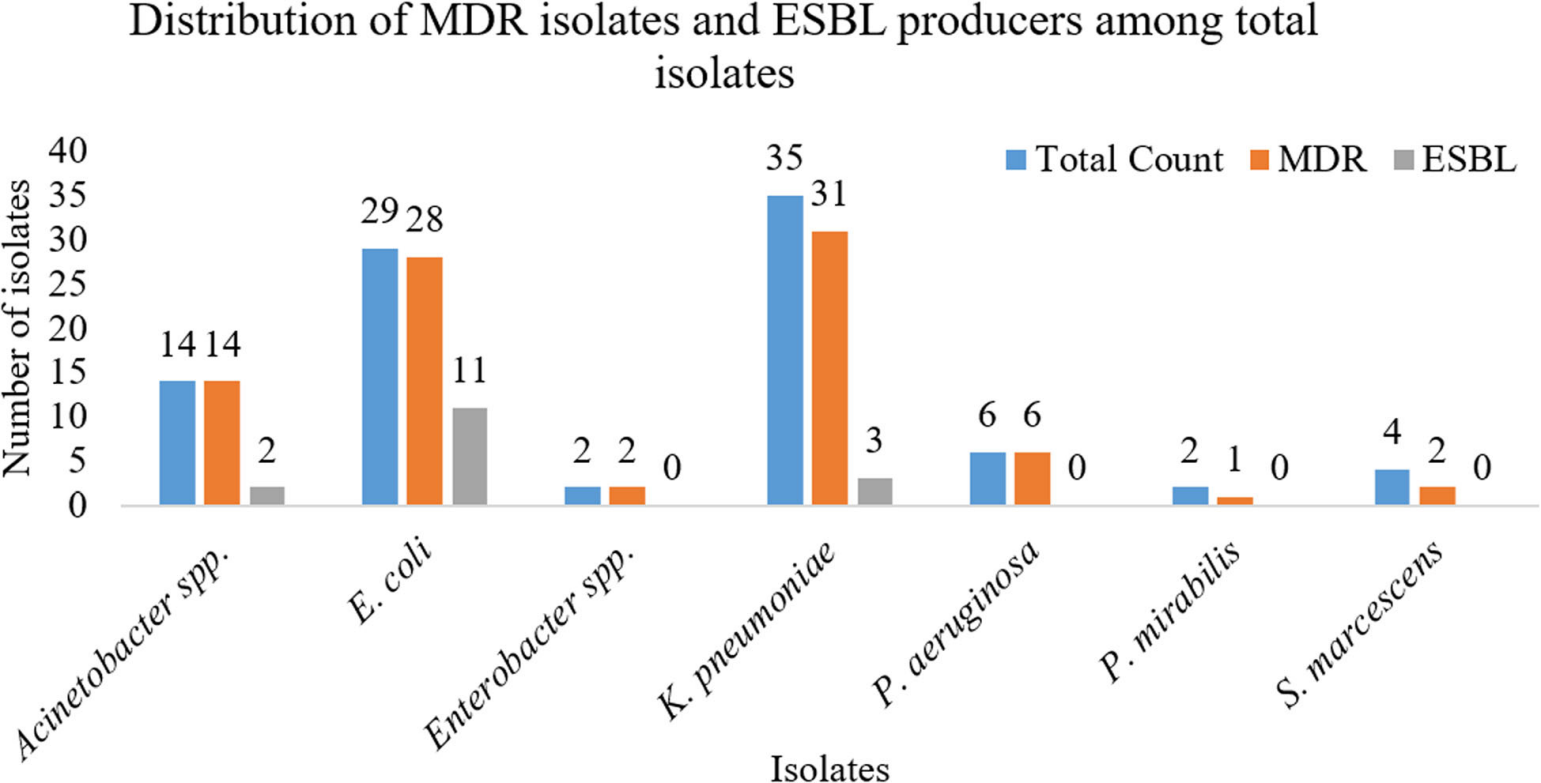
Distribution of MDR isolates and ESBL producers among total isolates

Among the 47 suspected isolates, 16 (34.0%) were found to be ESBL producers. ESBL production was the highest in *E*. *coli* (39.3%), followed by *Acinetobacter* and *K*. *pneumoniae*. *Pseudomonas aeruginosa*, *Enterobacter* spp., *Proteus mirabilis*, and *Serratia marcescens*, did not show any ESBL activity (Fig. 2).

### Demographic distribution of MDR isolates and ESBL producers

The prevalence of MDR was higher in males, whereas ESBL production was dominant in females. MDR prevalence was very high in all age groups. ESBL production was higher in the age-groups 31–40 years and 41–50 years. The percentage of MDR isolates was higher in outpatient than in inpatient, whereas ESBL production was higher in isolates from inpatient than the outpatient. Among the samples, MDR was seen very high, i.e., 80% or above, in isolates from all the samples except pus. ESBL producers were dominant in urine and absent in pus, wound, and tissue samples (Table 3).

**Table 3:** Demographic distribution of MDR isolates and ESBL producers

Characters	Growth positive, ***N*** (%)	MDR, ***N*** (%)	***p*** value	ESBL producers, ***N*** (%)	***p*** value
**Sex**					
Male (*n* = 424)	48 (11.3)	46 (95.8)	0.146	7 (14.6)	0.436
Female (*n* = 346)	44 (12.7)	38 (86.4)		9 (20.5)	
**Age group**					
< 10 (*n* = 123)	12 (9.8)	12 (100)	0.77	2 (16.7)	0.596
11–20 (*n* = 90)	4 (4.4)	3 (75)		0 (0.0)	
21–30 (*n* = 84)	9 (10.7)	8 (88.9)		1 (11.1)	
31–40 (*n* = 105)	21 (20.0)	20 (95.2)		5 (23.8)	
41–50 (*n* = 71)	8 (11.3)	7 (87.5)		3 (37.5)	
51–60 (101)	11 (10.9)	10 (90.9)		2 (18.2)	
> 60 (*n* = 196)	27 (13.8)	24 (88.9)		3 (11.1)	
**Department**					
Inpatient (*n* = 503)	33 (6.6)	27 (81.8)	0.016	7 (21.2)	0.693
Outpatient (*n* = 197)	59 (29.9)	57 (96.6)		9 (15.3)	
**Sample**					
Urine (*n* = 250)	52 (20.8)	47 (90.4)	0.195	12 (23.1)	0.714
Sputum (*n* = 185)	18 (9.7)	18 (100)		3 (16.7)	
Pus (*n* = 115)	6 (5.2)	4 (66.6)		0 (0.0)	
Blood (*n* = 166)	8 (4.8)	8 (100)		1 (12.5)	
Wound (*n* = 49)	5 (10.2)	4 (80)		0 (0.0)	
Tissue (*n* = 5)	3 (60.0)	2 (66.6)		0 (0.0)	
*n* = 770	*n* = 92 (11.9)	*n* = 84 (91.3)		*n* = 16 (17.4)	

## Discussion

In this study, 11.9% of the samples showed growth positivity. However, higher rates of growth positivity have been reported in similar studies in Nepal [33–36]. The major proportion of the sample in our study included the urine sample. Hence, the highest number of growths was observed in the urine sample and is parallel to the finding of Binod et al. [34] and Gurung et al. [37]. The high number of urine samples in our study might be because UTIs are the most common infections [38]. Although *E*. *coli* is the most common uropathogen [38], *K*. *pneumoniae* was the dominant isolate, followed by *E*. *coli* and *Acinetobacter* spp. in our study. Binod et al. [34], Aryal et al. [35], and Guragain et al. [39] reported a higher prevalence of *E*. *coli* followed by *K*. *pneumoniae*. Karn et al. [33] reported a lower prevalence of *K*. *pneumoniae* and *P*. *aeruginosa* than ours. Similar culture positivity was seen in both sexes; however, culture positivity was higher for females than males in the urine sample, and a higher prevalence of *E*. *coli* and *K*. *pneumoniae* was observed. Similar outcomes were drawn by Saderi et al. [40] and Yadav and Prakash [41]. Another study by Shrestha et al. [42] also unveiled *E*. *coli* as the most common organism. Higher culture positivity in urine samples from females might be attributed to the long urethra and proximity of urethral opening to the anus in females, increasing the chance of UTI in females [43].

More than 90% of the Gram-negative isolates showed multidrug resistance in this study. MDR was high in all the demographic characters studied. Most of the studies reported a lower MDR than this study [22, 44]. Panta et al. [45] have shown MDR rate in 100% of *Klebsiella* spp. and 80% of *Acinetobacter* spp. A study by Ghimire et al. [21] and Yadav et al. [46] recorded 96.8% and 82.5% of the isolates as MDR, respectively. Guragain et al. [39], Chakravarti et al. [47], and Sharma et al. [48] reported a lower MDR rate than ours in urinary *E*. *coli* and *K*. *pneumoniae* isolates. Surprisingly, in our study, the percentage of MDR was seen higher in outpatients than inpatients, which is in contrast to the report of Shrestha et al. [42]. The increasing rate of MDR in outpatients found in this study might be attributable to antibiotic abuse in Nepal such as non-empirical use, taking incomplete doses of antibiotics, and the easy availability of antibiotics without medical prescription.

ESBL producing bacteria are gradually increasing in hospital sectors, mostly as nosocomial infections, worldwide, and the occurrence of ESBL producing strains is changing rapidly over time with great variation [49]. In this study, ESBL producing bacteria was 17.4% of the total isolates. Kayastha et al. [22] and Raut et al. [50] reported a slightly higher prevalence of ESBL producing organisms, whereas Yadav and Prakash [41], Biswas et al. [51], and Afridi et al. [52] reported a much higher rate. ESBL production was slightly higher in females than in males, which parallels the findings reported earlier [49, 53]. In this study, a greater number of ESBL producers were encountered from inpatients (21.2%) than outpatients (15.3%). Similar findings have been reported by Mishra et al. [19] and Khanfar et al. [54]. In this study, the highest ESBL producer was found in urine (25.5%) followed by sputum (16.7%) and blood (12.5%) which is consistent with the findings of Luzzaro et al. [7], Parajuli et al. [20], and Khanfar et al. [54] where the major source of ESBL producers were urinary tract infections. However, Sharma et al. [48] reported a slightly lower and Guragain et al. [39] reported a slightly higher rate of ESBL producing bacteria among the urinary isolates. Over one third of *E*. *coli* was found to be the major ESBL producing isolate in this study, and this was similar to that reported by Parajuli et al. [20] and Ghimire et al. [21]. Yadav et al. [46] and Khanfar et al. [54] have also highlighted *E*. *coli* as the major ESBL producing bacteria. Kazemian et al. [55] reported higher ESBL production rates in *E*.*coli*, and *K*. *pneumoniae* than ours, whereas Sharma et al. [48] reported lower ESBL production in both strains. A study from Europe showed considerable variation, ranging from 1.6% (Latvia) to 23.2% (Russia), in the prevalence of ESBL-producing *E*. *coli* isolates [55]. The change in ESBL production pattern in bacteria seemed common as ESBLs were most often encoded on plasmids, which could easily be transferred between isolates [56, 57].

In this study, most of the first-line drugs were found to be ineffective. Antibiotics belonging to third- and fourth-generation cephalosporins, fluoroquinolones, amoxicillin, and amoxyclav were ineffective to ESBL producers and MDR strains. Khanfar et al. [54] have reported high resistance to gentamicin, amikacin, amoxicillin-clavulanic acid, and ciprofloxacin. However, our findings showed greater susceptibility to amikacin and gentamicin, which might be due to the minimal use of such drugs for treatment. On the contrary, the high degree of resistance to cephalosporins and fluoroquinolones in our study might be due to over-dependence on such antibiotics. Ogefere et al. [58] have also reported a high resistance against amoxicillin-clavulanate, ceftazidime, ceftriaxone, gentamicin, ciprofloxacin, and ofloxacin by ESBL producers. The overall antibiogram of Gram-negatives in this study showed a decreased susceptibility against most of the antibiotics, including carbapenems. This might be due to the haphazard use of the drug in hospital settings and unnecessary prescriptions by physicians before the arrival of actual culture and sensitivity reports. A higher sensitivity was seen for polymyxin B (100%) and carbapenems in ESBL producers in our study. Higher sensitivity to carbapenems was also reported by Luzzaro et al. [7], Mishra et al. [19], Ghimire et al. [21], Shrestha et al. [42], Biswas et al. [51], and Khanfar et al. [54] making them an ideal choice of drugs so far, for treating bacteria producing ESBL. All isolates showed sensitivity to polymyxin B. However, *K*. *pneumoniae*, *P*. *aeruginosa*, and *Acinetobacter* spp. showed greater resistance against most of the drugs except polymyxin B. All *E*. *coli* were sensitive to polymyxin B followed by imipenem. Polymyxin B and carbapenems seem to be the choice of drugs against ESBL producers and Gram-negative bacteria. However, these drugs should be considered alternatives until we have other effective drugs that could be administered safely. As the drug sensitivity pattern of different common Gram-negative bacteria seemed to change over time, a recent antibiogram for different Gram-negative isolates might help physicians treat bacterial infections [59].

## Conclusions

Regular surveillance of MDR and ESBL producers and implementation of hospital infection control policies to prevent the transmission of such isolates is much required. Polymyxin B and carbapenems seem to be the choice of drugs against ESBL producers and Gram-negatives but should be considered alternatives until we have other sensitive drugs that could be administered safely.

### Limitations

There were a few limitations to our study. Firstly, the sample size was around 770, which seemed significantly less than in other studies. The study was conducted within a short duration, i.e., 6 months. The data taken was purely obtained from only one hospital, which might not represent the whole population. Only a phenotypic study was performed. Studies on the molecular level would have strengthened the findings.

## Data Availability

The datasets generated and analyzed during the current study are available in the appropriate materials repository (tables and figures).

## Abbreviations

ESBL: Extended-spectrum beta-lactamase
CLSI: Clinical and Laboratory Standard Institute
MDR: Multidrug-resistant
BHI: Brain heart infusion
MHA: Muller Hinton agar
ATCC: American Type Culture Collection
*E*. *coli*: *Escherichia coli*
*K*. *pneumoniae*: *Klebsiella pneumoniae*
CI: Confidence interval
